# Spatio-Temporal Variability of Copepod Abundance along the 20°S Monitoring Transect in the Northern Benguela Upwelling System from 2005 to 2011

**DOI:** 10.1371/journal.pone.0097738

**Published:** 2014-05-20

**Authors:** Maya Bode, Anja Kreiner, Anja K. van der Plas, Deon C. Louw, Richard Horaeb, Holger Auel, Wilhelm Hagen

**Affiliations:** 1 BreMarE - Bremen Marine Ecology, Marine Zoology, University of Bremen, Bremen, Germany; 2 National Marine Information and Research Centre (NatMIRC), Swakopmund, Namibia; University of Connecticut, United States of America

## Abstract

Long-term data sets are essential to understand climate-induced variability in marine ecosystems. This study provides the first comprehensive analysis of longer-term temporal and spatial variations in zooplankton abundance and copepod community structure in the northern Benguela upwelling system from 2005 to 2011. Samples were collected from the upper 200 m along a transect at 20°S perpendicular to the coast of Namibia to 70 nm offshore. Based on seasonal and interannual trends in surface temperature and salinity, three distinct time periods were discernible with stronger upwelling in spring and extensive warm-water intrusions in late summer, thus, high temperature amplitudes, in the years 2005/06 and 2010/11, and less intensive upwelling followed by weaker warm-water intrusions from 2008/09 to 2009/10. Zooplankton abundance reflected these changes with higher numbers in 2005/06 and 2010/11. In contrast, zooplankton density was lower in 2008/09 and 2009/10, when temperature gradients from spring to late summer were less pronounced. Spatially, copepod abundance tended to be highest between 30 and 60 nautical miles off the coast, coinciding with the shelf break and continental slope. The dominant larger calanoid copepods were *Calanoides carinatus*, *Metridia lucens* and *Nannocalanus minor*. On all three scales studied, i.e. spatially from the coast to offshore waters as well as temporally, both seasonally and interannually, maximum zooplankton abundance was not coupled to the coldest temperature regime, and hence strongest upwelling intensity. Pronounced temperature amplitudes, and therefore strong gradients within a year, were apparently important and resulted in higher zooplankton abundance.

## Introduction

The Benguela Current upwelling region, extending along the South West African coast from 17 to 34°S, belongs to the four major eastern boundary currents in the world [1]. It is one of the most productive marine ecosystems reaching an average annual primary production of about 400–900 g C m^−2^ yr^−1^ [2]–[4]. Coastal upwelling of cold, nutrient-rich waters is a prerequisite for its high productivity, supporting large, economically important fish stocks, which have been severely exploited since the 1950s [1], [5]. As a unique feature, the Benguela Current upwelling system is bordered by warm, (sub-)tropical water masses, both at its northern and southern margins, i.e. by the Angola Current to the North and by the Agulhas Current and its retroflection zone to the South [6]. As part of the South Atlantic subtropical gyre, hypoxic, nutrient-rich South Atlantic central water (SACW) intrudes into the northern Benguela subsystem in summer, whereas oxygen-rich Eastern SACW is transported northwards during winter [7]. The permanent upwelling centre at Lüderitz (27–28°S) divides the Benguela ecosystem into a northern and a southern subsystem. Upwelling-favourable, south-easterly trade winds are most pronounced during spring and summer (September to March) in the southern Benguela Current (SBC) system and during winter and spring (July to November) in the northern Benguela Current (NBC) region [8]. The NBC region is characterized by highly variable upwelling intensities depending on local bathymetry, wind speed, wave action and frequent intrusions of tropical Angola Current waters [1]. Usually, the Angola-Benguela frontal zone (ABFZ) is situated between 14°S and 17°S, separating the oligotrophic tropical ecosystem from the nutrient-rich Benguela upwelling system [1]. The ABFZ is not a strong barrier, but periodically injects tropical waters into the surface layer of the NBC system [7], [9]. The central Namibian region (19–24°S) is often affected by onshore or alongshore flows of tropical waters from the North and oceanic waters from the West to Northwest [10]–[12].

As dominant primary consumers, zooplankton provide an important link from lower to upper trophic levels [13]. Copepods are the most abundant (55–95%) and diverse components of mesozooplankton communities in all marine regions [13]. In the NBC region they usually dominate zooplankton communities by 70–85% [14]. Thus, they play an important role in sustaining marine fish stocks, as they are the principal food source of sardines, anchovies and other pelagic fishes including their larval stages [2]. Long-term trends in, and relationships between, zooplankton, small pelagic fish stocks and environmental parameters have attracted growing interest in the context of climate change effects on marine ecosystems [15]. Due to its historical importance for Namibia's fishing industry, the area around Walvis Bay called “Walvis Bay routine area” (21–24°S) was studied most intensively in the past [16]–[18]. Between 1972 and 1982 the study area was expanded from 18 to 26°S during the South West African Pelagic Egg and Larval Surveys (SWAPELS) (for details see [19]). However, only a small fraction of these data have been analysed and published with regard to zooplankton, particularly copepod community composition [15].

The National Marine Information and Research Centre (NatMIRC) in Swakopmund, Namibia, is responsible for studying and managing the NBC ecosystem. In this context, they regularly monitor hydrographic parameters and plankton composition along several transects off the Namibian coast. Since March 2005, zooplankton samples were collected regularly along with oceanographic data, such as temperature, salinity, oxygen and chlorophyll *a* concentration in the upper 200 m along a transect at 20°S. This study provides the first analysis of these data with special reference to temporal and spatial variations in zooplankton abundance and copepod community structure from 2005 to 2011. The spatial and temporal distribution of total copepods as well as dominant calanoid copepod species was analysed and evaluated in relation to environmental factors.

## Materials and Methods

### Ethics Statement

Zooplankton samples have been collected by scientists from the National Marine Information and Research Centre (NatMIRC) in Swakopmund, which is part of the Ministry of Fisheries and Marine Resources and as such the responsible governmental agency for marine research in Namibia. This study on zooplankton does not include protected or endangered species. The deployment of WP2 nets has a negligible and non-measurable impact on the zooplankton community.

### Sampling

**Figure 1:**
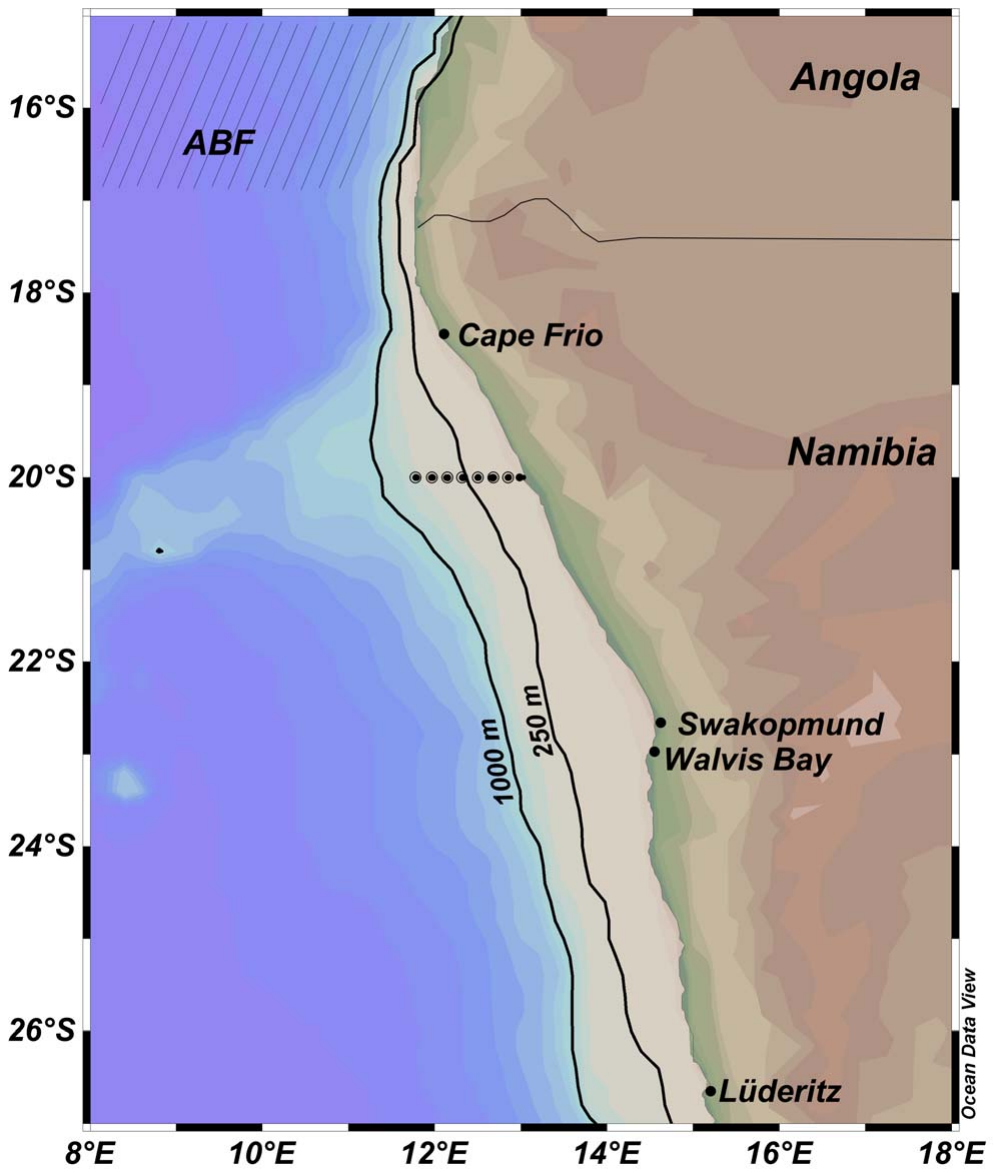
Location of the monitoring transect at 20°S off Namibia and approximate position of the Angola Benguela Front (ABF).

**Figure 2:**
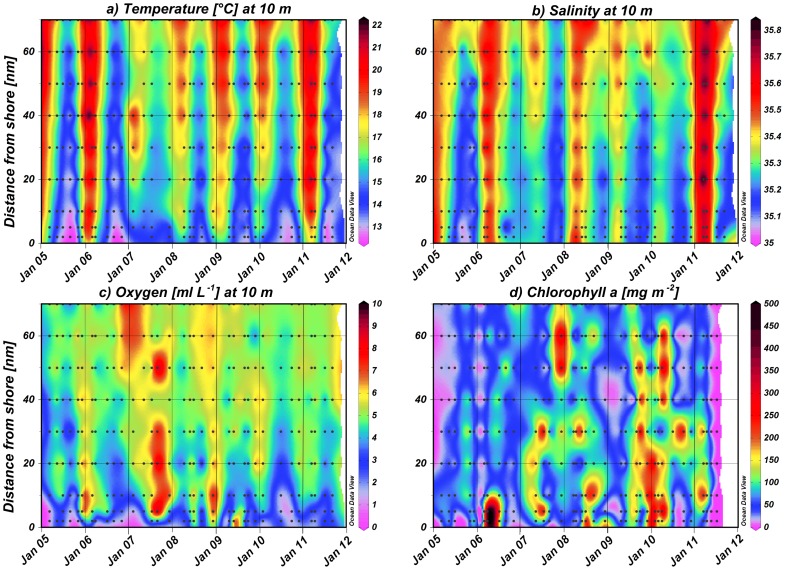
Horizontal and temporal distribution of a) temperature, b) salinity and c) oxygen concentration at 10 m and d) mean chlorophyll *a* concentration in the upper 30 m along the monitoring line at 20°S from March 2005 until September 2011. Black dots represent actual sampling positions and white areas indicate data gaps.

Zooplankton samples were collected along a transect at 20°S off Namibia approximately every other month starting in March 2005. The stations were always located at the same positions at 2, 5, 10, 20, 30, 40, 50, 60, and 70 nm from the coast (Fig. 1). For this study, stations within 5 nm from shore are referred to as “inshore stations”, while stations between 10 and 30 nm are on the continental shelf, thus “shelf stations”, and stations beyond 40 nm from the coast are referred to as “offshore stations”. Zooplankton samples were collected on board the research vessel “*Welwitchia*” using a WP2 net (0.25 m^2^ mouth opening, 200 µm mesh size) deployed from 200 m (or 10 m above the bottom if bottom depth <200 m) to the surface. The flow was measured by a calibrated flowmeter mounted within the mouth of the net. In addition, oceanographic data such as temperature, salinity and oxygen profiles were measured concomitantly with a conductivity temperature depth (CTD) sensor (Fig. 2). At each station chlorophyll *a* concentration was calculated with a Model 10 Turner design fluorometer from water samples collected at four depth levels between 2 and 30 m. A mean value was taken for each station and converted to surface chlorophyll *a* concentration (mg m^−2^). Zooplankton samples were preserved in 5% borax-buffered formaldehyde for subsequent taxonomic identification. Larger jelly-fish were removed from the samples before preservation. Mesozooplankton was identified and enumerated with special attention given to copepods. Several subsamples were taken using a Hensen Stempel pipette and analysed until about 500 individuals in total were counted from each station. Larger copepods (>1.5 mm) were identified to genus or even species level if possible and counted separately. Smaller species, including *Clausocalanus arcuicornis*, *Paracalanus parvus*, *Paracalanus crassirostris*, *Ctenocalanus vanus* etc., were combined under the category “small calanoid copepods”. Estimates of abundance per surface area (no. m^−2^) were calculated from the respective water volume filtered and maximum sampling depth. The total copepod abundances reported here include adult and copepodite stages CI-CVI. The sampling map as well as three dimensional figures were created with Ocean Data View 4 [20] using *DIVA gridding* as gridding method. A general linear model (GLM) was applied to explore the relationship of log transformed copepod abundances (totals and individual species) with several physical, temporal and spatial variables, but revealed no conclusive results.

### Principal component analysis

**Figure 3:**
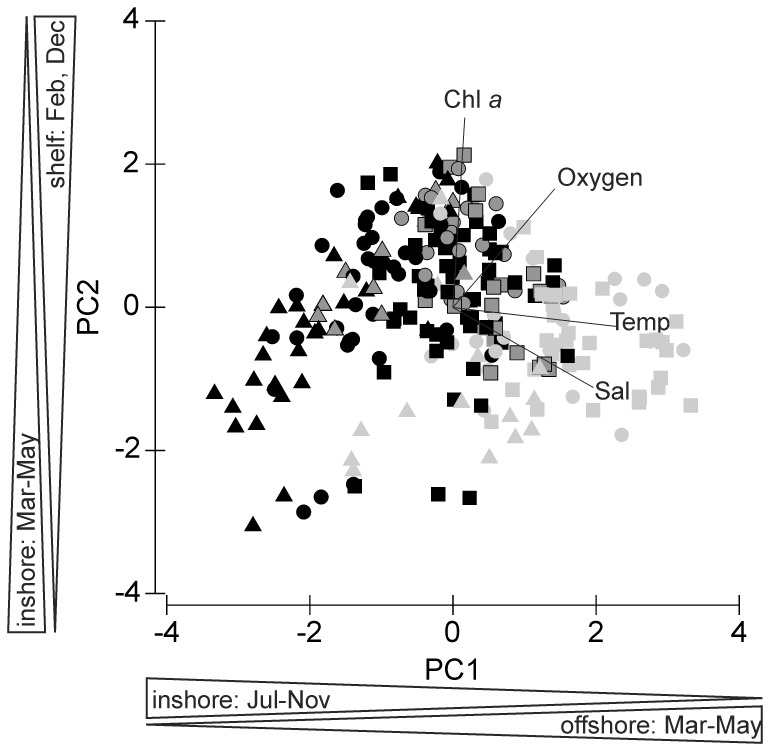
Inshore (triangles), shelf (circles) and offshore (squares) stations arranged according to the first two principal components. For a better overview, upwelling months (June-November) are black, while quiescent months (February to April) are light grey. Data of May and December are marked in dark grey, since these months showed a distinct oceanographic regime compared to the other months (start and end of upwelling period).

Variation in environmental data was explored applying principal component analysis (PCA) to identify environmental gradients throughout the study period. Potential environmental factors influencing copepod distribution were temperature, salinity, oxygen concentration (taken from the CTD data at 10 m depth) and chlorophyll *a*. Environmental parameters were standardized (log(x+1)) and normalized prior to PCA. Years were added as quantitative supplement, while the different months and sampling regions (inshore, shelf and offshore; for definition see above) were added as categorical supplement. The correlation matrix was used to calculate eigenvectors and principal components (PCs), which were ranked in order of significance. The contribution of each variable to each PC was estimated. In Figure 3, inshore, shelf and offshore stations are marked with different symbols (triangles, dots and squares, resp.), while months are pooled in quiescent (light grey: February-April), transition (dark grey: December and May), and upwelling (black: June-November) periods for a better overview (January was not monitored).

## Results

### Hydrography

**Figure 4:**
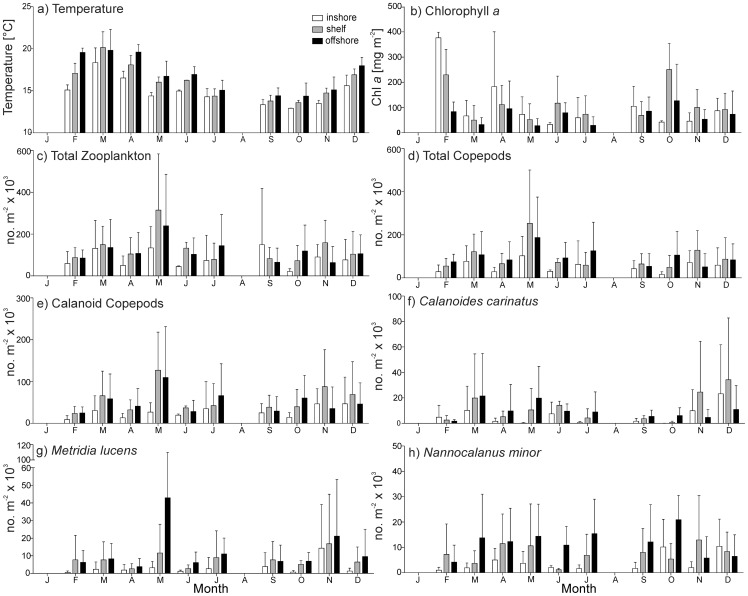
Monthly means (±SD) of a) temperature at 10 m, b) chlorophyll *a*, c) total zooplankton, d) total copepods, e) calanoid copepods, f) *Calanoides carinatus*, g) *Metridia lucens* and h) *Nannocalanus minor*. Note different scales.

**Figure 5:**
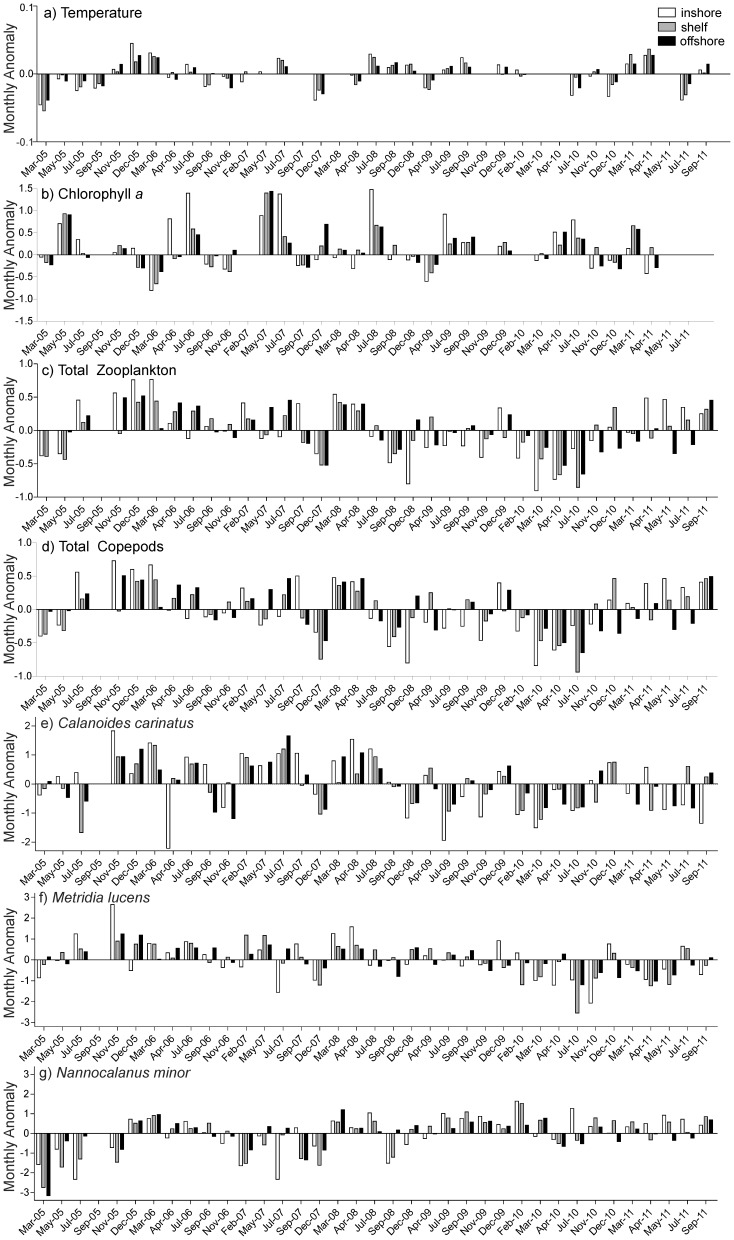
Log-transformed monthly anomalies between 2005 and 2011 of a) temperature at 10 m, b) chlorophyll *a*, and abundance (no. m^−2^) of c) total zooplankton, d) total copepods, e) *Calanoides carinatus*, f) *Metridia lucens*, and g) *Nannocalanus minor*.

At the 20°S transect, temperature and salinity showed a clear seasonal cycle with highest mean temperatures in March and lowest in September/October (Figs. 2 and 4). In March and April temperatures at 10 m depth ranged from 15° to 22°C. Coastal upwelling generally started in May or June with temperatures dropping below 15°C. From July to November temperatures were below 14°C, and coastal upwelling was strongest from September to November (<13°C). Cold, upwelled water was characterized by low salinities between 35.0 and 35.3, while maximum temperatures above 20°C were associated with salinities ≥35.7. Temperature generally increased from inshore to offshore stations by 3–4°C. However, there were substantial interannual variations in upwelling intensity and intrusions of tropical warm waters. Extensive warm-water intrusions of the Angola Current in late summer and autumn occurred in the same years as strong upwelling in spring. Therefore, years could be characterised according to the temperature amplitude with either high or low temperature gradients between spring and autumn. The years 2005/06 and 2010/11 showed comparatively wide temperature amplitudes (Figs. 2 and 5). In contrast, 2008/09 showed a rather weak gradient, with less intense upwelling in July and September 2008 accompanied by weak warm-water intrusions in April 2009 (Figs. 2 and 5). The years 2006/07, 2007/08 and 2009/10 cannot be clearly assigned to either of these two categories. Oceanographic data are missing in autumn 2007 and 2010, as well as in spring 2007 due to technical failure of the temperature and conductivity sensor of the CTD during these cruises. Because of these data gaps, the intensity of Angola Current intrusions in autumn 2007 is uncertain, but upwelling intensity was strong in spring 2006 and temperatures were below average in September and November 2006 (Figs. 2 and 5). In contrast, the upwelling intensity in spring 2007 was not recorded reliably, but the intrusion of tropical water in autumn 2008 was weak. However, temperatures in July 2007 were above average, whereas December 2007 and April 2008 were colder than usual (Fig. 5). Likewise, temperatures were above average in spring 2008 and 2009, thus, reflecting weaker upwelling intensity, whereas temperatures in April 2009 were below average (Fig. 5). However, the extent of warm-water intrusions was not recorded properly during these years; especially in autumn 2010 data are missing. Besides indicators of either weak upwelling or weak warm-water intrusions, inshore temperatures above 14°C each July from 2007/08 until 2009/10 may indicate years of low temperature gradients. In contrast, inshore temperatures below 14°C were measured in July 2005, 2006, 2010 and 2011, years of high temperature gradients.

Oxygen concentration was generally low within the first 10 nm on the shelf and the permanent oxygen minimum zone (OMZ) (<1.4 ml L^−1^) was more pronounced during quiescent months due to intrusions of low-oxygen Angola Current waters. The OMZ was usually observed between 40 and 60 m water depth, but oxygen concentrations below 1.4 ml L^−1^ even occurred at 10 m depth at 2 nm in March, September and November 2005, February 2007, April 2009 and November 2010 (Fig. 2). Chlorophyll *a* concentration in the upper 30 m was usually lower during the years 2005/06 and 2006/07, except for the peak value at 2 and 5 nm from shore in April 2006 of 540 mg Chl *a* m^−2^. From the year 2007/08 onwards maximum concentrations of >200 mg Chl *a* m^−2^ were measured on the shelf in July 2007, April, May and July 2008, September 2009 throughout April 2010, September 2010 and March/April 2011. In contrast, such high concentrations were present in the offshore region only in December 2007, September 2009 and April 2010 (Fig. 2). In general, chlorophyll *a* concentration was higher in the inshore or shelf region compared to the offshore region and showed highest monthly means in February and April (Fig. 4).

Principal component (PC) analysis identified three main components, which were determined by temperature, salinity, oxygen and chlorophyll *a* concentration and accounted for 95% of the variance in the environmental data set (Fig. 3). The first PC mainly represents temperature and salinity followed by oxygen concentration (p-values <0.0001) with positive eigenvectors (47% of variance explained). In the first categorical dimension the offshore stations in March, April and May had positive, whereas inshore stations in the upwelling months from July to November had negative effects (p-values <0.0001, except May: p-value  = 0.04). The second PC is mostly represented by chlorophyll *a*, oxygen concentration and salinity, for which chlorophyll *a* and oxygen content exhibited positive and salinity negative eigenvectors (p-values <0.0003; 28% of variance explained). Stations on the shelf in February and December were positively correlated with the second categorical dimension (p-values <0.004), while inshore stations in March, April and May showed negative effects (p-values <0.04). In Figure 3, inshore stations monitored from July to November coincided with colder temperatures and lower chlorophyll *a* concentrations, whereas inshore stations in December and May were associated with intermediate temperatures and higher oxygen and chlorophyll *a* concentrations. In contrast, inshore stations in February, March and April were characterized by warmer temperatures and lower chlorophyll *a* concentrations. In general, temperatures increased from inshore to offshore stations.

### Mesozooplankton community structure, abundance and distribution

**Figure 6:**
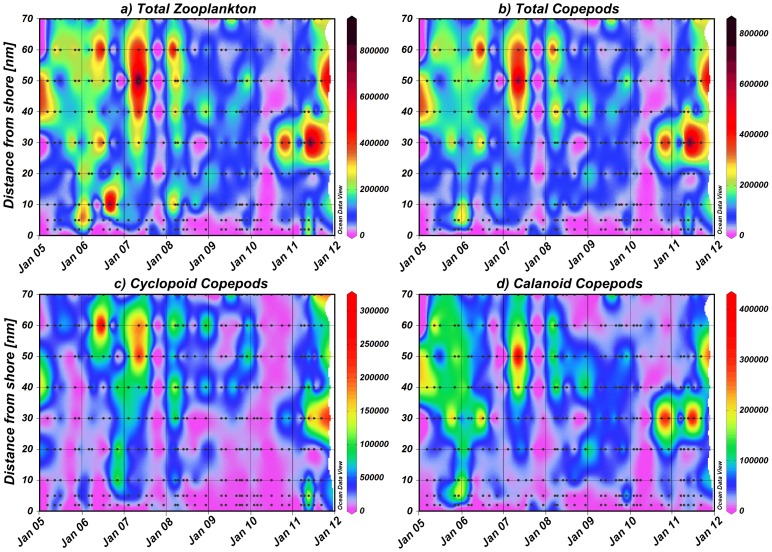
Abundance (no. m^−2^) and distribution of a) total zooplankton, b) total copepods, c) cyclopoid copepods and d) calanoid copepods shown for each year along the transect.

**Figure 7:**
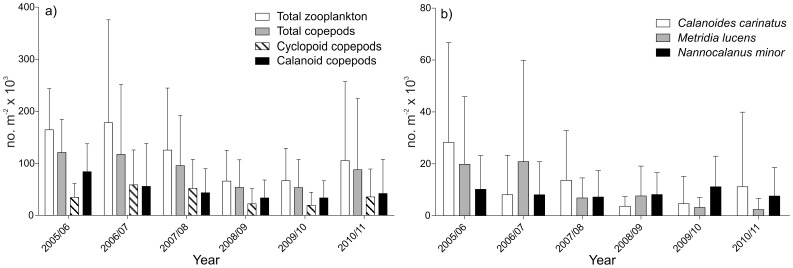
Mean annual abundance (no. m^−2^×10^3^±SD) of a) total zooplankton, total copepods, cyclopoid and calanoid copepods and the dominant species b) *Calanoides carinatus*, *Metridia lucens* and *Nannocalanus minor* for each year recorded.

**Figure 8:**
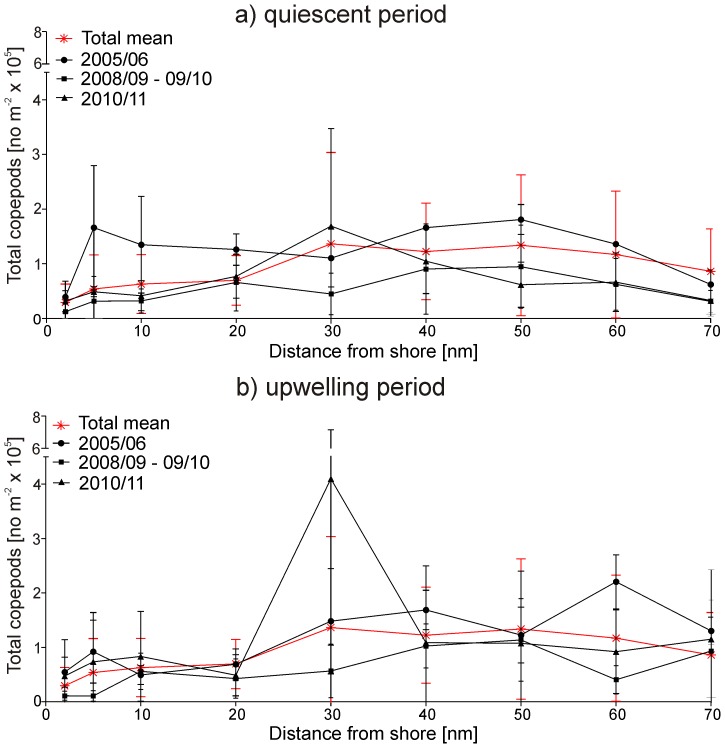
Mean abundance of total copepods (±SD) along the transect for upwelling (black) and quiescent (grey) months for the years 2005/06 and 2010/11 with extreme temperature gradients and the years 2008/09 and 2009/10 characterized by weak gradients. The total mean over all years was added (red).

Total mesozooplankton abundance ranged from 3×10^3^ m^−2^ at the 70 nm station in July 2010 to 943×10^3^ m^−2^ at 30 nm in May 2011 (Fig. 6). In 2005/06 and 2006/07, zooplankton abundance was highest (Figs. 6 and 7), while 2005/06 was characterized by a pronounced temperature gradient from spring to late summer (Figs. 2 and 5). In contrast, zooplankton abundance was lowest in 2008/09 and 2009/10 (Figs. 6 and 7), which were characterized by low seasonal temperature amplitudes. In 2010/11, upwelling intensity increased again, followed by massive warm-water intrusions in autumn and high zooplankton abundances, particularly at 30 nm from shore (Figs. 6 and 7). Copepods usually dominated the zooplankton community and, on average, accounted for 78% of total zooplankton abundance. Hence, the distributional pattern of total copepods reflected that of total zooplankton (Figs. 4–7). The only exceptions were observed at the 10 nm station in September 2006 and March 2008, when echinoderm larvae dominated the zooplankton catch and between 2 and 10 nm from shore in May 2011, when cyclopoid copepods prevailed (Fig. 6). Apart from 2010/11, mean annual copepod abundance was generally higher in the offshore region, but was not significantly different from mean abundances on the shelf (Mann-Whitney U test, p-values ≥0.054). In contrast, annual copepod abundance averages were significantly lower at the 2 and 5 nm stations in the year 2006/07, 2008/09 and 2009/10 compared to the shelf and offshore stations in the same years (Mann-Whitney U test, p-values ≤0.018). In general, total copepod densities increased from inshore towards offshore regions with maxima usually occurring between 30 and 50 nm, albeit with strong interannual variations (Figs. 4, 6 and 8). Extremely high copepod densities were recorded at 30 nm from shore in September/December 2010 and July/September 2011 explaining the maximum value in Figure 8.

Total copepod abundance ranged from 3×10^3^ m^−2^ at 70 nm from the coast in July 2010 to 857×10^3^ m^−2^ at 30 nm in May 2011. Maxima of more than 500×10^3^ m^−2^ were encountered in May 2007 and 2011 as well as in July 2006. Copepod densities of 300–500×10^3^ m^−2^ occurred between March and May 2005, 2007, 2008, 2011, in July 2006, 2007, 2011 and in September 2011 as well as in November-December 2005 and 2010. No obvious seasonal cycles were observed throughout the year. Abundances were usually highest in November and December and in March and May (Fig. 4), but the pattern varied from year to year. On average, the copepod community was dominated by calanoid copepods (56%), 40% belonged to cyclopoid copepods (of which 79% were *Oithona* spp. and 19% *Oncaea* spp.) and 4% were harpacticoid copepods. Maximum numbers of calanoid copepods reached 432×10^3^ m^−2^ at 50 nm from shore in May 2007 and 338×10^3^ m^−2^ at 30 nm in July 2011.

### Calanoid community structure, abundance and distribution

#### Dominant species

**Figure 9:**
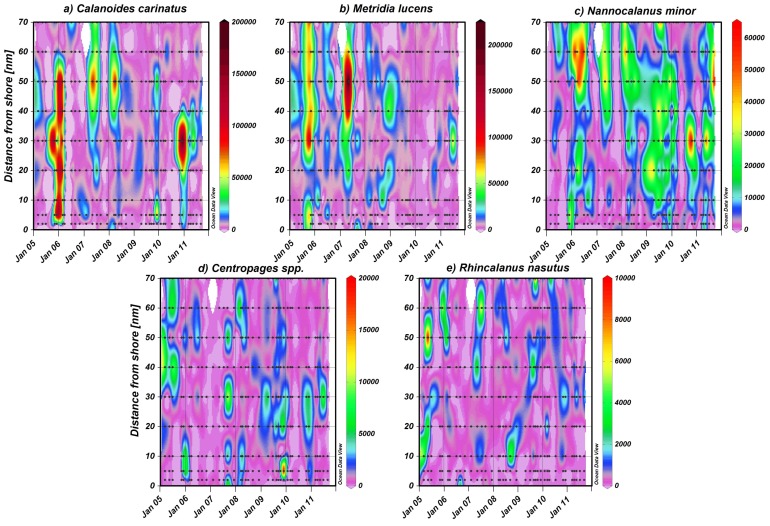
Monthly abundances (no. m^−2^) of the dominant copepods a) *Calanoides carinatus*, b) *Metridia lucens*, c) *Nannocalanus minor,* d) *Centropages* spp. and e) *Rhincalanus nasutus* along the transect 20°S between March 2005 and September 2011. Note different scales.

**Figure 10:**
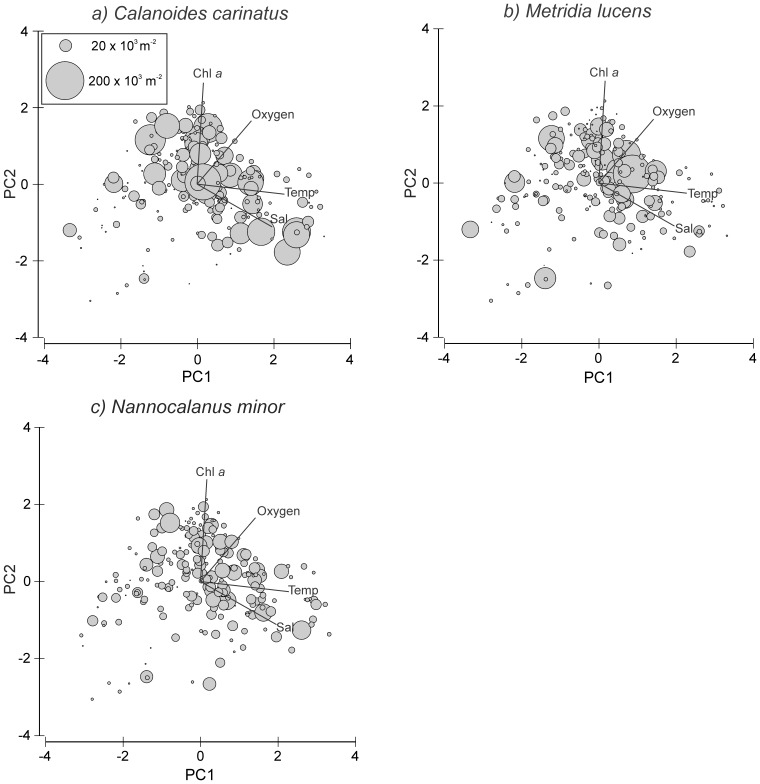
Abundances (no. m^−2^) of a) *Calanoides carinatus*, b) *Metridia lucens*, and c) *Nannocalanus minor* for each station along the first two principal components PC1 and PC2 as shown in Fig. 3. The area of each circle is proportional to the abundance shown in the legend.

**Figure 11:**
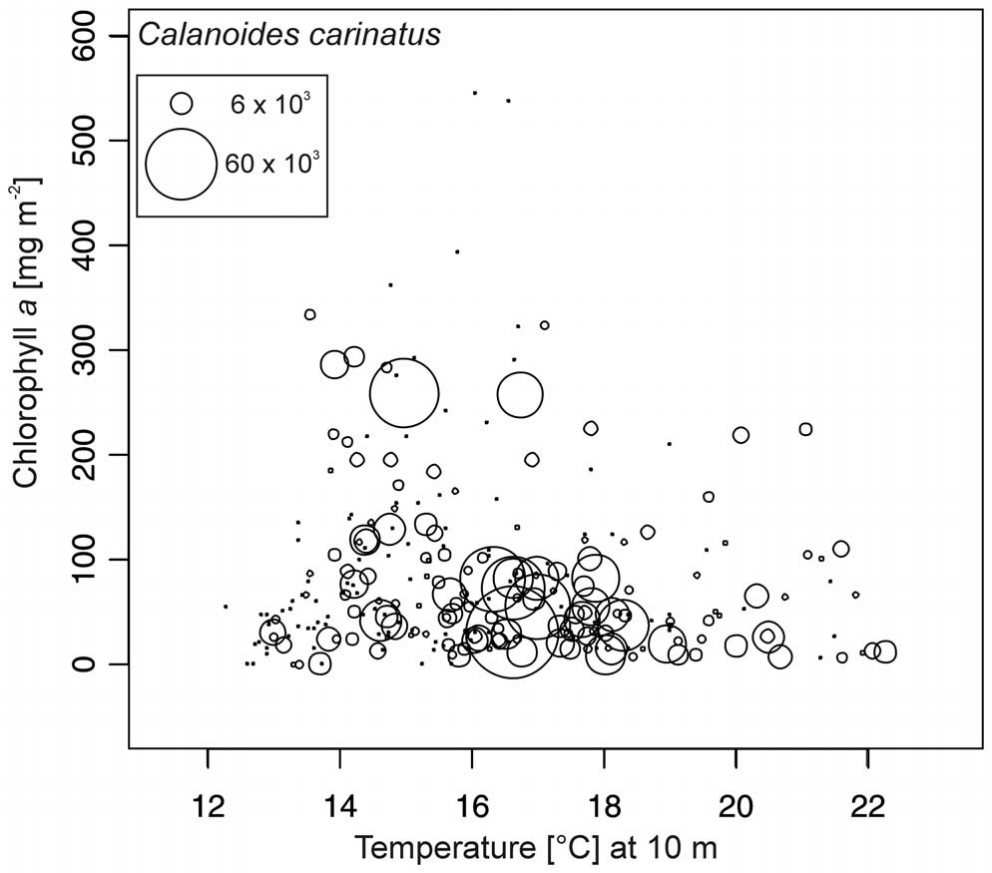
Abundances (no. m^−2^) of *Calanoides carinatus* in relation to chlorophyll *a* concentration and temperature at 10 m depth. The area of each circle is proportional to the abundance shown in the legend.

Among the larger calanoid copepods, *Calanoides carinatus, Metridia lucens* and *Nannocalanus minor* clearly dominated. Spatial and temporal distribution patterns of the dominant and consistently abundant calanoid species are shown in Figure 9. The majority of *C. carinatus* was observed in patches between 10 and 50 nm from shore. Maximum numbers of 100–180×10^3^ m^−2^ were observed inshore and on the shelf in December 2005, 2010 and in March 2006. High abundances between 20 and 90×10^3^ m^−2^ were concentrated at 30–50 nm from shore in March 2005, April 2008, May 2007, 2011, November 2010 and at 5 nm from shore in December 2009. *C. carinatus* occurred regularly in large patches until the end of 2007/08 (Figs. 5 and 9). Annual abundance was highest in 2005/06, whereas the population decreased in 2008/09 and 2009/2010 and increased again from November 2010 onwards (Fig. 7). Generally, higher abundances across the transect were found in March and December as well as inshore and on the shelf in November, but also in the shelf and offshore region in May (Fig. 4). Maximum numbers occurred mainly during November and December, when temperatures were intermediate and chlorophyll *a* concentrations were high (Figs. 10 and 11). In some years high abundances also occurred in March (especially 2006 and 2008), when temperatures were high and chlorophyll *a* had already decreased (Figs. 10 and 11). During upwelling months (July to October), densities of *C. carinatus* were generally lower (Fig. 4).

The majority of *Metridia lucens* was found between 20 and 50 nm from the coast (Fig. 9). Maximum abundance of 223×10^3^ m^−2^ was found 50 nm from shore in May 2007, whereas abundances of 50–100×10^3^ m^−2^ were recorded at 30 nm in November 2005 and July 2011, as well as at 40 nm in December 2008. Highest annual abundances were observed in 2005/06 and 2006/07. From 2007/08 onwards annual densities decreased, and in 2009/10 and 2010/2011 *M. lucens* was almost absent (Figs. 7 and 9). Only in July 2011 higher abundance occurred again at 30 nm from the coast (Figs. 5 and 9). No clear seasonal cycle was observed, but highest densities along the transect were usually observed in November and in the offshore region in May (Fig. 4). Highest abundances generally coincided with higher chlorophyll *a* concentrations and intermediate temperatures. However, higher densities also occurred during intensive upwelling in July and September, and throughout March and April, the warmest months (Fig. 10).

*Nannocalanus minor* was much more evenly distributed during the monitoring period (Fig. 7). Maximum abundances were present in the years 2005/06 and 2009/10, but mean annual abundance was more or less stable throughout the years (Figs. 7 and 9). Before 2007/08, the majority of the population was found between 40 and 70 nm from shore, whereas in 2008/09 and 2009/10 *N. minor* mainly occurred between 20 and 50 nm, which were years characterized by low temperature gradients between spring and late summer. From 2010/11 onwards, highest densities were caught at 30 nm and further offshore. In general, monthly abundances were higher offshore, except for November, December and February, when mean abundances were elevated on the shelf (Fig. 4). Maximum abundance of 62×10^3^ m^−2^ was recorded at 30 nm from the coast in November 2010, while abundances between 50 and 60×10^3^ m^−2^ were found at 50 to 60 nm from shore in March, April and July 2006. High densities of 20–50×10^3^ m^−2^ co-occurred with warm-water intrusions in December 2005, 2008, 2010, February 2010 and March and April 2008 (Figs. 2 and 9). However, such high numbers of *N. minor* were also observed during upwelling months in July 2006, May and July 2007, July 2008, May-November 2009, and July and September 2011. Abundances were quite evenly distributed across the first two principal components without evidence of a seasonal cycle (Fig. 10).

#### Less abundant species

The distribution of *Centropages* spp. was patchy throughout the entire monitoring period except for the year 2006/07, when they were almost absent (Fig. 6). The majority of *Centropages* spp. was found between 20 and 50 nm from shore. However, maximum abundance of 20×10^3^ m^−2^ was recorded at 5 nm from shore in December 2009. High abundances of 11×10^3^ m^−2^ occurred at 30 nm in September 2007, while 9×10^3^ m^−2^ were recorded around 40 nm from March-July 2005 and at 30 nm in July 2011. No clear seasonal cycle was observed. Generally, abundance was highest during upwelling months each year, and in some years in December (2005, 2009, 2010), when upwelled water was warming up. However, high densities also occurred between February and April (2005, 2006, 2008, 2009, 2010), when temperatures were higher but still ≤19.5°C.

The distribution of *Rhincalanus nasutus* was very patchy between 10 and 50 nm from shore throughout the whole monitoring period (Fig. 6). Maximum abundance of 10×10^3^ m^−2^ occurred at 50 nm from shore in May 2005. Abundances between 3 and 6×10^3^ m^−2^ were recorded on the shelf in March and May 2005 and September 2008 as well as offshore in December 2005, May and July 2007 and September 2009. From December 2009 onwards, densities did not exceed 3×10^3^ m^−2^. *R. nasutus* was associated with cooler, upwelled water from May to November each year and in some years with stratifying water masses in December (2005, 2008 and 2010). However, higher abundances were also recorded in March 2005, 2006, 2008 and 2010, when surface temperatures were high, but usually still below 20°C (only >20°C in March 2006).

## Discussion

The Benguela Current is a highly productive eastern boundary current upwelling system [19]. Due to high seasonal and interannual variability in wind field structure and thus upwelling intensity, as well as strength and directions of ocean currents, the structure and dynamics of plankton communities are complex and may only be understood by continuous long-term monitoring [21]. In contrast to the southern Benguela Current system (SBC), plankton data are still scarce for the northern Benguela Current region (NBC), especially considering long-term dynamics [15]. This paper presents the first detailed study of a monitoring programme of temporal and spatial variability in zooplankton abundance and copepod community structure along a transect at 20°S, where at certain times the influence of tropical Angola Current waters can be strong [22]. Spatial, interannual and seasonal variations in copepod abundance and community structure were analyzed in context with changes of the environmental conditions.

### Hydrographic regime

Hydrographic patterns reflected the general annual upwelling cycle in the northern Benguela Current region off Namibia, exhibiting a strong seasonal cycle with maximum temperatures in mid to late austral summer and lowest temperatures in mid to late spring [23], [24]. This seasonality correlates with the general wind pattern with lowest velocities of southerly winds and a stronger westerly component in January and February, whereas highest wind velocities were generally recorded in October [23]. In the present study lowest temperatures (≤13°C) and salinities (≤35.3) were recorded inshore from July to November indicating maximum coastal upwelling, with increased chlorophyll *a* concentrations across the shelf during this time, except for spring 2009, when extremely high chlorophyll *a* values were also measured beyond the shelf. May and December were generally characterized by intermediate temperatures and salinities as well as high chlorophyll *a* concentrations. In contrast, March and April were the warmest months with highest temperatures and salinities, but lower chlorophyll *a* concentrations. During austral summer (December-March), oligotrophic waters from the Angola Current intrude into the northern Benguela region, whereas during late winter and spring (July-September) cool, nutrient-rich waters may extend from the Benguela Current far north into Angola waters enhancing primary production [25].

### Copepod abundance and distribution

With an average of 78%, copepods represented the most abundant component within the mesozooplankton community, which is in line with previous studies in the NBC [14], [22]. Maximum copepod abundance was observed at 30 nm from shore in May 2011 reaching 857×10^3^ m^−2^, while typical values ranged from 300–500×10^3^ m^−2^, which is similar to published data [14], [22], [26]. In October 1979, maximum abundance of 700×10^3^ m^−2^ was estimated at a cross-shelf transect between 20–21°S to 100 nm offshore using a WP-2 net [26]. In April 1986, total copepod abundances of 300–800×10^3^ m^−2^ were recorded off Walvis Bay, while 100–300×10^3^ m^−2^ were collected further north at 20°S using a multiple opening-closing rectangular midwater trawl (1 m^2^ mouth opening) [22]. In December 2000, a maximum of 520×10^3^ m^−2^ was reported at 20 nm off Walvis Bay (23°S) (WP-2 net with 200 µm mesh size; [14]).

Highest copepod abundances were generally observed between 30 and 50 nm from shore on the 20°S transect. This peak in zooplankton abundance over the shelf break and above the continental slope is in line with observations further south at 25° S [27]. However, in the present study, high abundances were also observed on the shelf during December, whereas lowest abundances generally occurred inshore from July to November. This agrees with observations made around Walvis Bay in 2000 [14], where highest abundances of copepods occurred around 40 nm from July to November and between 10 and 30 nm in December. Strikingly low densities of copepods were recorded within upwelling water masses at inshore stations from July to November [14]. Between 20 and 21°S, low abundances occurred closer inshore, increasing in the shelf region and peaking between 70–100 nm from shore between October and November 1979 [26]. In the present study, the coldest water masses occurring inshore from September to November were also depleted in copepods. In contrast, warmer water masses that had been transported further away from shore, exhibited higher copepod abundances during upwelling months. This is in line with the generally observed succession process from initial upwelling via phytoplankton blooms to subsequent zooplankton development [15]. Maximum phytoplankton abundance usually occurs two days after an upwelling event followed by increasing copepod abundance about 20–23 days after initial upwelling [26]. For example, the exceptionally low number of copepods in December 2007 may be explained by a delay of the upwelling period in this year. Temperatures in July 2007 were above and in December 2007 below average throughout the whole transect, while chlorophyll *a* concentration was unusually high in the offshore region in December 2007. Our study emphasises that zooplankton abundance is highly variable in space and time. This pronounced variability with its multiple peaks during each year seems to be affected by the influence of Angola Current water as well as by fluctuating, diffuse upwelling processes, which are often disrupted by plumes and filaments and have been observed especially in the NBC [14], [22].

Total copepod abundance in the NBC was lower at the near-shore stations (usually 30×10^3^ to 100×10^3^ ind m^−2^) than in the Humboldt Current Upwelling System off Chile (150×10^3^ to 300×10^3^ m^−2^, [28]), where total copepod abundance was strongly dominated by the neritic species *Paracalanus parvus* (10×10^3^ to 100×10^3^ m^−2^) and most of the species showed high abundances at the very near-shore stations [28]. In contrast to the Humboldt upwelling system, there was no pronounced cross-shelf gradient in total copepod abundance off Namibia [28]. The biomass species *Calanus chilensis*, however, occurred in numbers of several hundred to thousand individuals per square meter off northern Chile [28], also exhibiting lower abundances at the very near-shore stations during the upwelling season [29], similar to the ecologically comparable *Calanoides carinatus* off northern Namibia.

### Interannual variability and seasonal cycles

Our study indicates a decline in copepod abundance for the years 2008/09 and 2009/10. This decrease coincided with low spring to late summer temperature gradients, thus, less pronounced upwelling in spring and only moderate warm-water intrusions in late summer (Figs. 2 and 6). In contrast, years with strong upwelling in spring and extensive warm-water intrusions in later summer, as in 2005/06 and 2010/11, were characterized by high copepod abundances suggesting a strong link between zooplankton distribution and physical forcing. By contrast, zooplankton abundance was positively correlated to upwelling intensity in the SBC [30]. A 100-fold increase in mesozooplankton abundance was reported in the SBC between 1950 and 1995 [30], while upwelling intensities also increased during this time period [31]. Since 1995 zooplankton densities, particularly those of larger copepods, have decreased again in the SBC, accompanied by an increase in anchovy biomass [15], [30]. In contrast to the SBC, the NBC has not been studied regularly making long-term assessments difficult to date [14], [16], [18], [21], [22], [26], [27], [32], [33]. However, studies in the NBC indicate that zooplankton abundance decreased slightly from the 1950s to the early 1980s, whereas a long-term increase in zooplankton abundance was observed from 1983 onwards [15]. Total copepod densities increased 6-fold along a 70 nm transect off Walvis Bay (23° S) between 1978 and 2004 followed by a decline after 2005 [15], [25]. Recently, contradictory long-term trends have been observed in the major upwelling regimes of the world. For example, macrozooplankton abundance decreased severely off southern California during the last half century, while sea surface layers warmed by 1.5°C and stratification increased [34], [35]. However, no long-term decrease of calanoid copepods was observed off southern California from 1951 to 1999, while the calanoid species composition in spring remained stable over the entire monitoring period, except for six anomalous years [35].

The seasonal signal of total copepod abundance in the present study was similar to previously observed patterns, although much more diffuse and variable from year to year, despite the evident seasonal upwelling cycle. Maxima of total copepod abundance were mainly recorded in November and December (2005, 2008, 2009, 2010) and in March and May (2005, 2006, 2007, 2011). However, in 2006 and 2007 peaks were also observed in July. No direct correlation between upwelling intensity (temperature), phytoplankton activity (chlorophyll *a*) and zooplankton abundance emerged, which is in line with previous observations in the Walvis Bay region [15]. In the SBC at St. Helena Bay (32°S), a clear seasonal signal of zooplankton abundance was observed with a maximum abundance in late summer followed by a sharp decline in autumn [36]. Contrarily, in the “Walvis Bay routine area” (21–24°S), two annual peaks of zooplankton abundance were observed during a three year period in the 1950s: A primary peak from November to December in late spring to early summer, and a secondary peak from March to June during late summer and autumn [18], which corresponded to peaks in maximum phytoplankton concentrations [16]. In contrast, low copepod densities were observed off Walvis Bay from March to June in the year 2000, whereas abundances increased from July to December in the same year, indicating that seasonal cycles in the NBC are not as obvious as in the SBC [14].

Seasonal variations in abundance of the dominant copepods were also less pronounced in the Humboldt Current system [37]. Off southern California, most of the dominant copepods exhibited higher abundances in spring during the upwelling season than in winter, whereas *Metridia pacifica*, the second most dominant copepod in this region, occurred in comparable numbers throughout the two seasons [35]. Even during a 27 day time series at St Helena Bay in the southern Benguela Current system, daily changes of zooplankton biomass appeared to be uncorrelated with upwelling cycles [38]. Our study further clarifies, that fluctuations in abundance of most species cannot be explained by upwelling processes alone, but that behavioural responses (e.g. diel vertical migrations) and biological interactions (e.g. predation and mortality) need to be considered to fully understand life cycles and distributional patterns of copepods in upwelling regions [37]–[39]. To disentangle these complex relationships, a much higher temporal resolution of biological and oceanographic data is suggested for future monitoring in the NBC region, including process studies of shorter duration with sampling coverage of days to weeks [38].

### Copepod community structure

Long-term changes in copepod community structure and size composition have been observed for the southern Benguela system [30] and have been indicated for the NBC off Walvis Bay [14]. The eight dominant species described for the NBC were *Centropages brachiatus*, *Calanoides carinatus*, *Metridia lucens*, *Nannocalanus minor*, *Clausocalanus arcuicornis*, *Paracalanus parvus*, *Paracalanus crassirostris* and *Ctenocalanus vanus* [14], [30]. Earlier studies in surface waters (100–0 m) of the “Walvis Bay routine area” in 1962–63 reported *P. crassirostris* and *Paracartia africana* as strictly neritic species, while *C. carinatus*, *P. parvus*, *M. lucens* and *C. brachiatus* dominated the cool-water community on the shelf in order of decreasing abundance (N70 net, 195 µm mesh size) [16], [18]. In contrast, *N. minor* was most abundant in offshore warm-water communities [18]. Between 1985 and 1990, the four dominant species in Namibian waters were *C. carinatus*, *R. nasutus*, *M. lucens* and *C. brachiatus* in order of decreasing abundance (180 µm mesh size) [21], [27], while in 2000 *M. lucens* prevailed over *Rhincalanus nasutus* (200 µm mesh size) [14]. In the present study area further north off Walvis Bay at 20°S, community composition was highly variable between years and months, but seven calanoid species always dominated the shelf and offshore region. The three larger species competing for the first rank were *C. carinatus, M. lucens* and *N. minor* followed by *Centropages* spp., *Calocalanus* spp. and, depending on the month, *Eucalanus hyalinus*, *Candacia* spp. and *Rhincalanus nasutus*. Off Oregon, in the California Current system, on- and off-shelf variations in copepod biomass and community structure reflected the origins of the source water currents while the copepod assemblage could be divided into a nearshore and a midshelf to outer shelf group [40]. In our study, no such clear zonation pattern was identified.

In general, *C. carinatus* prevailed especially on the shelf in March, November and December, whereas *M. lucens* and particularly *N. minor* were more evenly distributed and did not show seasonal patterns. Around Walvis Bay, *C. carinatus* exhibited higher abundances inshore between June and August at the onset of upwelling and peaked during October and December at the end of the upwelling season [14]. Species-specific temperatures (<13°C) and low salinity preferences were identified for *C. carinatus* characterizing recently upwelled waters [12]. Hence, this species was referred to as cold-water species [14], [27]. However, our study does not show such a clear distributional pattern due to strong interannual variations (Fig. 9). Abundances of *C. carinatus* were generally high in May at the onset of the upwelling season and in November and December at the end of the upwelling season, but also in March during the quiescent season (Fig. 4). However, this pattern was not consistent over the years, e.g. extremely low densities occurred in December 2007 which may be due to delayed upwelling during this year. During upwelling months, high abundances of *C. carinatus* were correlated with intermediate temperatures and higher chlorophyll *a* concentrations above the shelf break and further offshore, which characterize stabilizing and sun-warmed offshore-moving, water masses sometime after the actual upwelling event (Figs. 10 and 11). In December, *C. carinatus* also occurred inshore and on the shelf, while temperatures were intermediate and chlorophyll *a* levels high. In March and April, *C. carinatus* was associated with high temperatures but lower chlorophyll *a* concentrations.

In contrast to previous studies mentioned above, *N. minor* represented one of the three dominant species. In the years 2008/09 and 2009/10, when *C. carinatus* and *M. lucens* populations had decreased, *N. minor* dominated the calanoid community. These years were characterised by weaker upwelling allowing stronger mixing with oceanic waters, which could explain the consistently higher densities of *N. minor* during this period. *N. minor* is usually considered a warm-water species with low abundances off Walvis Bay, where this species was associated with intrusions of warm oceanic waters onto the shelf in periods of weak upwelling [18], [27], [41]. During upwelling months, *N. minor* occurred only in low abundance at the most oceanic stations (160 nm) off Walvis Bay [41]. In contrast, in the northwesternmost part of the study area around Walvis Bay (∼22°S), *N. minor* took over the dominant role in the copepod community [18]. During the Benguela Niño in 1963, when unusually high sea surface temperatures and salinities moved southwards from Angola into the northern Benguela region, abundances of *N. minor* increased considerably [17]. Along the monitoring line at 20°S, the influence of Angola Current water is even more pronounced than around Walvis Bay and generally higher abundances of *N. minor* are expected [19], [22].

These observations underline that zooplankton dynamics in the NBC are highly complex and do not follow well-defined patterns. The present study elucidates that plankton dynamics in this region are driven by non-linear interactions between wind, upwelling events, solar radiation, regeneration and recycling of nutrients, biological interactions and species-specific response and development times of the organisms [15], which exacerbates attempts to model such a highly variable ecosystem. It emphasises the necessity of long-term studies with consistent high-resolution monitoring and analyses of the driving forces and key species to disentangle their interactions and dynamics. This is particularly important in order to understand ecosystem functioning and predict future changes with potentially severe impacts on such highly productive ecosystems.

## References

[1] Boyer D, Cole J, Bartholomae C (2000) Southwestern Africa: Northern Benguela Current region. Mar Pollut Bull 41(1–6): 123–140.

[2] Heileman S, O'Toole MJ (2008) Benguela Current LME. In: Sherman K, Hempel G, editors.The UNEP Large Marine Ecosystem Report: A perspective of changing conditions in LMEs of the world's regional seas UNEP Regional Seas Report and Studies No 182 United Nations Environment Programme. Nairobi, Kenya. pp. 100–142.

[3] Monteiro PMS (2010) The Benguela Current System. In: Liu K-K, Atkinson L, Quinones R, Talaur-McManus L, editors.Carbon and nutrient-fluxes in the continental margins.Berlin: Springer. pp. 65–78.

[4] Brown PC, Painting SJ, Cochrance KL (1991) Estimates of phytoplankton and bacterial biomass and production in the northern and southern Benguela ecosystems. S Afr J mar Sci 11: 537–567.

[5] Crawford RJM, Shannon LV, Pollock DE (1987) The Benguela ecosystem part VI. The major fish and invertebrate resources. Oceangr Mar Biol Rev 25: 323–505.

[6] Shannon LV, Agenbag JJ, Buys MEL (1987) Large- and mesoscale features of the Angola-Benguela front. S Afr J Mar Sci 5(1): 11–34.

[7] Mohrholz V, Bartholomae CH, van der Plas AK, Lass HU (2008) The seasonal variability of the northern Benguela undercurrent and its relation to the oxygen budget on the shelf. Cont Shelf Res 28: 424–441.

[8] Shannon LV, O'Toole MJ (2003) Sustainability of the Benguela*: ex Africa semper aliquid novi*. In: Hempel G, Sherman K, editors.Large marine ecosystems: trends in exploitation, protection and research. Amsterdam: Elsevier.

[9] Shannon LV, Hempel G, Malanotte-Rizzoli P, Moloney CL, Woods J (2006) Benguela: Predicting a Large Marine Ecosystem. Large Marine Ecosystems 14. Amsterdam: Elsevier. 410 p.

[10] Boyd AD, Salat J, Masó M (1987) The seasonal intrusion of relatively saline water on the shelf off northern and central Namibia. S Afr J mar Sci 5: 107–120.

[11] Lass HU, Mohrholz V, Nausch G (2000) Hydrographic and current measurements in the area of the Angola-Benguela front. J Phys Oceanogr 30: 2589–2609.

[12] O'Toole MJ (1980) Seasonal distribution of temperature and salinity in the surface waters off South West Africa. 1972-74. Investl Rep Sea Fish Inst South Africa 121–125.

[13] Longhurst AR (1985) The structure and evolution of plankton communities. Prog Oceanogr 15: 1–35.

[14] Hansen FC, Cloete RR, Verheye HMV (2005) Seasonal and spatial variability of dominant copepods along a transect off Walvis Bay (23°S), Namibia. Afr J Mar Sci 27(1): 55–63.

[15] Hutchings L, Verheye HMV, Hugget JA, Demarcq H, Cloete R et al. (2006) Variability of plankton with reference to fish variability in the Benguela Current Large Marine Ecosystem - An overview. In: Shannon LV, Hempel G, Malanotte-Rizzoli P, Moloney CL, Woods J, editors.Large Marine Ecosystems. Amsterdam: Elsevier.

[16] Kollmer WE (1963) The pilchard of South West Africa (*Sardinops ocellata*). Notes on zooplankton and phytoplankton collections made off Walvis Bay. Investl Rep mar Res Lab S W Afr 78 pp.

[17] Stander GH, De Decker AHB (1969) Some physical and biological aspects of an oceanographic anomaly off South West Africa in 1963. Invest Rep Div Sea Fish S Afr 81: pp 46.

[18] Unterüberbacher HK (1964) The pilchard of South West Africa (*Sardinops ocellata*). Zooplankton studies in the waters off Walvis Bay with special reference to the Copepoda. Investl Rep mar Res Lab S W Afr 11: 42 pp (+ Plates 42–36).

[19] Shannon LV, Pillar SC (1986) The Benguela ecosystem. Part III. Plankton. In: Barnes M, editor.Oceanography and Marine Biology An annual review.Aberdeen: University Press. pp. 65–170.

[20] Schlitzer R (2013) Ocean Data View 4, http://odv.awi.de.

[21] Timonin AG (1992) Zooplankton and environmental variability in the northern Benguela upwelling area. Russ J Aqua Ecol 1(2): 103–113.

[22] Olivar M-P, Barangé M (1990) Zooplankton of the northern Benguela region in a quiescent upwelling period. J Plankt Res 12(5): 1023–1044.

[23] Stander GH (1962)The pilchard of South West Africa (*Sardinops ocellata*). General hydrography of the waters off Walvis Bay, South West Africa 63 p.

[24] Stander GH (1964) The Benguela Current of South West Africa. 43 p. +77 figures.

[25] Hutchings L, van der Lingen CD, Shannon LJ, Crawford RJM, Verheye HMS, et al (2009) The Benguela Current: An ecosystem of four components. Prog Oceanogr 83: 15–32.

[26] Postel L, Arndt EA, Brenning U (1995) Rostock zooplankton studies off West Africa. Helgoländer Meeresunters 49: 829–847.

[27] Timonin AG (1991) Correlation between distribution of zooplankton and hydrologic conditions in the Benguela upwelling zone. Oceanology 31(2): 181–195.

[28] Escribano R, Hidalgo P (2000) Spatial distribution of copepods in the north of the Humboldt Current region off Chile during coastal upwelling. J Mar Biol Ass UK 80: 283–290.

[29] Escribano R (1998) Population dynamics of *Calanus chilensis* in the Chilean eastern boundary Humboldt Current. Fish Oceanogr 7(3/4): 245–251.

[30] Verheye HMV, Richardson AJ, Hutchings L, Marska G, Gianakouras D (1998) Long-term trends in the abundance and community structure of coastal zooplankton in the southern Benguela system, 1951-1996. S Afr J mar Sci 19: 317–332.

[31] Shannon LV, Crawford RJM, Pollock DE, Hutchings L, Boyd AD, et al (1992) The 1980s - A decade of change in the Benguela ecosystem. In: S Afr J Mar Sc Payne AIL, Brink KH, Mann KH, Hilborn R, editors. Benguela trophic functioning. 12: 271–276.

[32] Barangé M (1989) Zooplankton size structure off Namibia in July 1983 and 1984. Colln scient Pap int Comm SE Atl Fish 16(1): 31–41.

[33] Brenning U (1985) Structure and development of calanoid populations (Crustacea, Copepoda) in the upwelling regions off North West and South West Africa. Beitr Meeresk 53: 3–33.

[34] Roemmich D, McGowan J (1995) Climatic warming and the decline of zooplankton in the California Current. Science 267: 1324–1327. 17812604 10.1126/science.267.5202.1324

[35] Rebstock GA (2002) Climatic regime shifts and decadal-scale variability in calanoid copepod populations off southern California. Global Change Biol 8: 71–89.

[36] Verheye HM, Hutchings L, Huggett JA, Painting SJ (1992) Mesozooplankton dynamics in the Benguela ecosystem, with emphasis on the herbivorous copepods. In:Benguela trophic functioning. Payne, AIL, Brink, KH, Mann, KH and R Hilborn (Eds.). S Afr J mar Sci 12: 561–584.

[37] Hidalgo P, Escribano R (2007) Coupling of life cycle of the copepods *Calanus chilensis* and *Centropages brachiatus* to upwelling induced variability in the central-southern region of Chile. Prog Oceanogr 75: 501–517.

[38] Verheye HM (1991) Short-term variability during an anchor station study in the southern Benguela upwelling system: Abundance, distribution and estimated production of mesozooplankton with special reference to *Calanoides carinatus* (Krøyer, 1849). Prog Oceanogr 28: 91–119.

[39] Peterson WT, Arcos DF, McManus GB, Dam H, Bellatoni D, et al (1988) The nearshore zone during coastal upwelling: Daily variability and coupling between primary and secondary production off Central Chile-. Prog Oceanogr 20: 1–40.

[40] Morgan CA, Peterson WT, Emmett RL (2003) Onshore-offshore variations in copepod community structure off the Oregon coast during the summer upwelling season. Mar Ecol Prog Ser 249: 223–236.

[41] Timonin AG, Arashkevich EG, Drits AV, Semenova TN (1992) Zooplankton dynamics in the northern Benguela ecosystem, with special reference to the copepod *Calanoides carinatus* . S Afr J Mar Sci 12(1): 545–560.

